# A Case of the TOF with APV Complicated with Polyhydramnios and Severe Bronchomalacia

**DOI:** 10.1155/2016/3641453

**Published:** 2016-08-07

**Authors:** Ali Seven, Emine Esin Yalinbas, Rahmi Ozdemir

**Affiliations:** ^1^Department of Obstetrics and Gynecology, Dumlupinar University School of Medicine, 43040 Kutahya, Turkey; ^2^Department of Pediatrics, Dumlupınar University School of Medicine, 43040 Kutahya, Turkey; ^3^Department of Pediatric Cardiology, Dumlupınar University Kutahya Evliya Çelebi Training and Research Hospital, Kutahya, Turkey

## Abstract

Absent pulmonary valve syndrome (APVS) is a rare congenital heart disease with severe pulmonary insufficiency, characterized with aneurysmal dilation in the pulmonary artery and one or both of its branches. We presented a rare case with APVS and literature review in this letter. Prenatal USG examination of the fetus at the 26th week of gestation revealed severe polyhydramnios, dilatation at right ventricle, and abnormal appearance of the heart. At the 31st gestational week, the baby was born with cesarean section. The newborn had right heart failure but had no hydrops fetalis. Therefore, severe respiratory distress observed in the infant has been associated with pulmonary complications. The infant, who had respiratory acidosis according to blood gas analysis, was intubated and attached to mechanical ventilator. Despite progressively increased respiratory support and other interventions, the infant died on the 3rd day of admission. Compression against bronchial tree and esophagus due to dilated pulmonary artery and its branches may inevitably lead to bronchomalacia and polyhydramnios. In conclusion, presence of polyhydramnios and the possibility of severe bronchomalacia should be kept in mind; and due to the risk of early neonatal mortality, delivery should be performed in a center where pediatric heart surgery is available.

## 1. Introduction

Absent pulmonary valve syndrome (APVS) is a rare congenital heart disease with severe pulmonary insufficiency, characterized with aneurysmal dilation in the pulmonary artery and one or both of its branches, along with a rudimentary or dysplastic pulmonary valve [1]. Absence of pulmonary valve may accompany ventricular septal defect (Fallot type AVPS), or it may be together with intact ventricular septum and tricuspid atresia (non-Fallot type APVS) [1]. It is different from TOF regarding its clinical findings and course and hemodynamic features. The abnormal dilation in the pulmonary artery (PA) causes compression against the tracheobronchial tree, which can lead to extensive bronchomalacia or other various pulmonary complications [2]. In this paper, we present a case of Fallot type APVS diagnosed in antenatal period which ended up with early neonatal death.

## 2. Case Report

**Figure 1:**
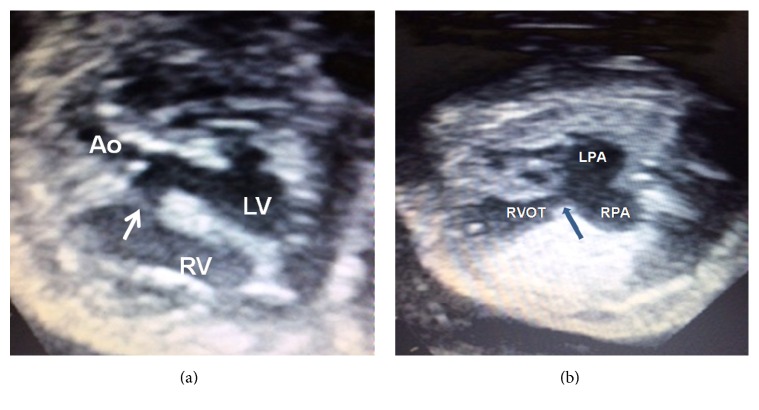
(a) This sagittal image reveals a large subaortic ventricular septal defect (white arrow) between the right ventricle (RV) and the left ventricle (LV). Aorta originating from the left ventricle is 50% dextroposed. (b) A coronal image of the fetal thorax reveals the aneurysmally dilated left pulmonary artery (LPA) and right pulmonary artery (RPA). Arrow indicates absence of pulmonary valve. RVOT: right ventricle outflow tract.

Twenty-seven-year-old woman was having her fourth pregnancy; prenatal USG examination at the 26th week of gestation revealed severe polyhydramnios, dilatation at right ventricle, and abnormal appearance of the heart. In fetal echocardiographic examination, there was a pulsatile, cystic structure in the neighborhood of the heart. There was subaortic large ventricular septal defect (VSD), and the aorta originating from the left ventricle was 50% dextroposed (Figure 1(a)). Right ventricle was opening into a giant pulmonary artery and its branches, which appeared pulsatile and cystic in structure. The pulmonary artery had clover type morphological appearance (Figure 1(b)). The pulmonary valve was dysplastic and rudimentary. Color Doppler sonography showed severe pulmonary insufficiency accompanying the stenotic physiology. The pulmonary artery peak systolic velocity, tissue Doppler derived myocardial performance index (Tei index), and peak early right ventricle filling velocity (*E*)/peak late atrial filling velocity (*A*) ratio were 246.8 cm/s, 0.59, and 0.48, respectively. The fetus had no hydrops fetalis. Based on these findings, absent pulmonary valve syndrome with tetralogy of Fallot was considered in the fetus. At the 30th gestational week, the patient was hospitalized due to preterm labor. Steroid treatment was administered along with tocolysis. At the 31st gestational week, the infant was born with C/S. After birth, the infant showed peripheral cyanosis and intercostal retractions and had respiratory acidosis according to blood gas analysis. The infant was intubated and attached to mechanical ventilator. Since the chest X-ray had an appearance that was consistent with respiratory distress syndrome (RDS), surfactant therapy was administered along with mechanical ventilatory support. Additionally, positive inotropic support was administered due to presence of hypotension. The infant had continuing respiratory distress; therefore, repeated surfactant applications were performed. The infant had arterial oxygen saturation levels at about 70%, and peripheral circulation was poor. High frequency oscillatory ventilation (HFOV) was initiated on the 2nd day of admission. Despite progressively increased respiratory support, the infant died on the 3rd day of admission.

## 3. Discussion

Absent pulmonary valve syndrome (APVS) is a rare congenital heart defect characterized by absent or rudimentary pulmonary valve leaflets. APVS is associated with airway compression which continues to remain a challenge. Compression against bronchial tree and esophagus due to dilated pulmonary artery and its branches may inevitably lead to bronchomalacia and polyhydramnios [3]. In their series including 21 fetal cases with absent pulmonary artery, Volpe et al. detected 22q11 microdeletion in 25% of the cases [4]. However, there was no phenotypical feature in our case to suggest presence of this microdeletion. Also, the same study reported frequent prevalence of bronchomalacia associated with cardiomegaly and dilated pulmonary artery and reported that this condition was an important prognostic factor indicating bad prognosis [4]. Our case had serious respiratory distress that necessitated intubation in the delivery room, which was linked to severe bronchomalacia and prematurity. In their series with 9 antenatally diagnosed cases, Kawazu et al. detected polyhydramnios in 3 patients; they reported “balloon type PA” morphology in dilated pulmonary arteries in those three cases. In their study, all of the hydramniotic cases were lost during early neonatal period. It has been reported that cases with “clover type PA” morphology had more favorable course compared to the balloon type [5]. Our case had clover type pulmonary artery morphology and was polyhydramniotic. During the followup, the case was lost at the postnatal 72nd hour despite increased respiratory support.

It has been stated in many studies that pulmonary artery wall thickness would be greater in the presence of patent ductus arteriosus (PDA) and that this would prevent development of balloon type PA, resulting in a better prognosis [4]. Although our case did not have PDA, the PA morphology was clover type; however, our case had a bad clinical course as it is expected in the absence of PDA. Indeed, not all APVS cases with clover type PA morphology have good prognosis unless the anomaly is corrected with cardiac surgery at early period of life. In our case, Doppler echocardiography findings during fetal life were not severe, and there was no hydrops fetalis; therefore, it was not foreseen that pulmonary complications after birth would be so severe; hence, we thought urgent cardiac surgery would not be necessary in our case. If the infant's overall condition allowed after cesarean section, we would have transferred the infant to a center where the infant can undergo the emergent cardiac operation. Also, prematurity and neonatal period are the risk factors for early mortality following that kind of operation [6]. In a recent study, Yong et al. shared their 38 years of experience in that kind of surgery with 52 patients. According to the results of their study, early mortality occurred in 2 (66.7%, 2 of 3) neonates and 5 (20.8%, 5 of 24) infants, whereas there were no mortalities (0%, 0 of 25) among patients older than 1 year at time of operation [6].

In conclusion, while presence of polyhydramnios and hydrops fetalis, absence of PDA, and balloon type PA morphology are poor prognostic indicators, it should also be kept in mind that clover type PA morphology may be associated with bad prognosis as well. Correct antenatal diagnosis in these cases can be made with echocardiography at midsecond trimester. In case of polyhydramnios, the possibility of severe bronchomalacia should be kept in mind; and due to the risk of early neonatal mortality, delivery should be performed in a center where pediatric heart surgery is available.
